# The Efficacy of Diode Laser Palatoplasty on Patients with Troublesome Snoring

**DOI:** 10.22038/ijorl.2020.39316.2299

**Published:** 2020-11

**Authors:** Mohammed-Radef Dawood, Nihad-Fouad Shukri

**Affiliations:** 1 *Department* *of Otolaryngology, Mustansiriyah University, College of Medicine, Baghdad, Iraq.*; 2 *Department of Otolaryngology, Al-Yarmouk Teaching Hospital, Baghdad, Iraq.*

**Keywords:** Diode laser, Palatoplasty, Snoring

## Abstract

**Introduction:**

Simple Snoring is low frequency sound without obstructive sleep apnea, produced mainly from vibration of the upper airway wall in the soft palate; it can be bothersome and may causes social problems. So the aim of this study is an attempt for relieving simple snoring though, stiffening the soft palate via transoral diode laser palatoplasty.

**Materials and Methods:**

Forty six adult patients with socially troublesome snoring were recruited in this stuy. Adequately history was taken to rule out witnessed evidence of obstructive sleep apnea. Then full otolaryngological examination, with soft palate vibration grading assessment by Muller maneuver were followed, consequently the eligible patients were underwent transoral 810 nm diode laser palatoplasty under local anesthesia and followed-up 6 months post-operatively.

**Results:**

The loudness of subjective snoring assessed by visual analogue score (VAS) was relieved from 6.6 to 2.4 and by Muller maneuver was improved from 2.7 to 1.9, and for the frequency from 2.1 Hz to 0.3 Hz, and from 2.4 Hz to 0.55 Hz for the duration pre to post-operative with P value of 0.75 to 0.023 respectively, So the overall frequencies of improvement of snoring loudness results had revealed a total improvement (no snoring) were detected in 4 patients, and moderate improvement had been found in 30 patients, while mild improvement of snoring had been seen in 10 patients, however about 2 patents got no benefit from the surgery.

**Conclusion:**

Stiffening of the soft palate by diode laser had shown to be significantly beneficial in relieving the snoring intensity as it had a positive impact on its subjective loudness, frequency and duration.

## Introduction

Snoring is a common complaint that increasingly recognized as a socially troublesome problem that had impact on patients and their family life, especially as it serves as an important known risk factor for development of sleep apnea and cardiopulmonary consequences, epidemiological studies have shown that it occurs in about 20% of adults, and about 50% of those found in age above 60 years (1). It is a sound of a low frequency generated from vibration of the upper airway walls due to partial upper airway obstruction that mainly detected in the soft palate, however, in approximately 30 % of them may also be present at other sites such as base of the tongue, epiglottis, and also the tonsils. Simple or primary snoring is defined as snoring without the evidence of obstructive apnea (OSA), or a frequent arousals or abnormalities of gas exchange. Aggravating factors may include alcohol consumption, smoking, and obesity as high basal metabolic index (BMI), male gender and older age (2). 

Definition of apneic status is clinically assessed by the following criteria; symptoms and signs of OSA, apnea/hypopnea syndrome, nocturnal choking, witnessed apneic incidents during sleep, daytime sleepiness, morning headaches or sensation of morning heavy head without alcohol consumption in the previous night, Epworth sleepiness score≥ 15, and (BMI) ≥ 28 kg/m2 (3). Indications of surgery for simple snoring were patients with socially problematic habitual snoring without evidences of (OSA) were the obstruction is localized at one level in the upper airway mainly at palatal level, or even multi-segmental obstruction predominately at palatal level (2). 

 Surgical therapy for primary snoring continues to evolve. There are various surgical techniques have been applied to the soft palate which aimed to improve its stiffness by increasing its tension, via reducing its compliance, so this thought to increase the intrinsic strength of the soft palate so it will able to resist the big pressure changes that happened within the nasopharynx during strenuous exercise, as this aimed to improve the soft palate function (4). 

Uvulopalatopharyngoplasty (UPPP) was the first palatal procedure performed for the treatment of snoring, it continues to be an option but not frequently used because of the need for a general anesthetic and significant morbidity associated with recovery, in case of primacy snoring on the other hand minimally invasive techniques as is addressed in the outpatient clinical setting with would be ideal, its advantages over UPPP included the fact that it could be done in the office under local anesthetic with a lower incidence and severity of complications, since then, less invasive and more tolerable procedures have been evolving with a goal of simple, durable, and with lasting effects, so the goal is reduce the severity and duration of snoring that is bothersome and potentially harmful without subjecting the patient to too much risk, several minimal invasive palate surgical techniques have been used for relieving for primary snoring such as; radiofrequency tissue volume reduction / thermal ablation, injection palatoplasty, pillar soft palatal implants, and anterior palatoplasty, laser palatoplasty (5). 

The real tissue stiffening mechanism of the laser is not clearly understood, were it appears to be observed in two phases, an acute mucosal shrinking tissue response, that observed immediately following laser radiation, which is a common thermal effects laser-tissue interaction phenomenon, then a delayed phase, which believed to be a corner-stone in the laser stiffening procedure, as a gradual "hardening" tissue generated by submucosal fibroblasts (6). 

A variety of medical applications now a day used diode laser for the following reasons; compact size, radiance, precision and increased power in conveying the uttermost performance, these significant medical applications are photodynamic therapy (PDT), aesthetics, and many other surgical fields, as it offer many remarkable advantages such as a bloodless operating field, minimal tissue swelling and less scarring and even barely noticed post-surgicalpain (7). The use of the 810-nm diode laser as the treatment of choice for oral soft tissue therapy is reliable because we obtained acceptable healing of the lesions with minimal adverse effect. Thus, in oral soft tissue surgery, the use of 810-nm diode lasers may be the best choice (8). So this study was aimed to evaluate the efficiency of stiffening of the soft palate via tansoral interstitial diode laser in relieving simple snoring. 

## Materials and Methods 

A prospective interventional study enrolled 46 adult patients with socially problematic snoring, from January 2017 to January 2018. An informed consents and Institutional Review Board committee were approved, as well as the hospital record numbers.

The patients needed to meet the following criteria to be participated in the study; age more than 18 years of both genders, bothersome snoring Stanford as visual analogue scale (VAS)>3, Epworth sleepiness score<11, basal metabolic index (BMI)<30, neck circumference <43 cm, and neither smoking or alcoholism, while any patient with following criteria will be excluded such as; patient with witnessed apnea and any symptoms and signs of OSA, nasal or pharyngeal pathology or previous surgery, any surgical history for snoring, patient any patient with craniofacial abnormalities, patient with uncontrollable gag reflux, also uncooperative patients and those whom not attended regular follow up, and any patient with psychological problems confirmed by his medical report.

The informative data were included the following clinical parameters; 

Demographic information that included: age, gender, occupation, and marital status, then the anthropometric measurements body mass index (BMI) calculated by the following this question: BMI = weight per KG / (height per meter) 2, and also the neck circumference (NC) was measured through the examination of the circumference of the neck at the level of the superior border of the cricothyroid membrane with the patient in the upright position by tape measure, also a precise history of snoring that explained by bed partner or/and roommate which included the following data which were evaluated as ; 

a. The intensity or loudness of snoring was evaluated through Stanford visual analogue Scale (VAS) for subjective snoring, as the following; grade 0 means no snoring, from 1-3 means mild (dose not disturb the partner during sleep), from 4-6means loud snoring (enough to disturb the partner), from 7-9 means very high (disturbs people in other room), while if 10 it means intense (the partner leave the room).

 b. The frequency and the duration of the snoring incidence was scored as the following: score 0 means it is very rare or even none, score 1 it means some nights (<50%), score 2 it means most nights ( > 50% ), while score 3 it means every or whole the nights.

 c. The duration of snoring was assessed as the following: grade 3 means whole the night, if grade 2 was > 50% of the night it means most of the night, while if grade 1 it was < 50% of the night it means some of the night ang grade0 means it was hardly or even none.

 d. Clearness of any snoring-associated symptoms like morning headache, witness apnea, chocking at night or excessive day Time sleepiness that evaluated by Epworth sleepiness scale as following; 0 = never doze, 1 = Slight chance of dozing, 2 = Moderate chance of dozing, 3 = High chance of dozing as shown in table 1.

**Table 1:** Epworth sleepiness scale

	**Situation**	**0**	**1**	**2**	**3**
1	Reading				
2	Television watching				
3	Public place (theatre or meeting) inactive sitting				
4	An hour transport passenger without a break				
5	Afternoon rest lying down				
6	Talking to someone				
7	After lunch "without alcohol" sitting quietly				
8	Five minutes vehicle sitting blocked in traffic				
	Total				

Then all patients were subjected to proper otolaryngological examination, and the degree of the soft palate vibration grading was assessed by the means of the retropalatal obstruction via the Muller maneuver pre and postoperatively. Muller maneuver technique. The procedure was done in attempt to confirm and grading the retropalatal obstruction by introducing a fiber optic nasopharyngoscope (optim 2.7 mm) to level of hypophayrnx at first in sitting position then in supine position, the patients was tried to inhale with a maximal inspiratory efforts against closed mouth and sealed nose ''reverse to valsalva maneuver" the nasopharyngoscope was withdrawn slowly at the level of passavants ridge. The degree of the soft palate collapse was evaluated as the following: grade 0 means no obstruction, grade 1 were 0- 25% obstruction, grade 2 were 25- 50%, grade 3 were 50-75%, grade 4 were 75-100% obstruction.

Cephalometry was done to try excluded any craniofacial abnormalities with maxillofacial consultation. Then all the eligible patients were underwent trans oral Diode laser palatoplasty under local anesthesia.

*Operative technique*

The Patient was sat on comfortable ENT chair. Topical 10% Lidocaine was sprayed onto the oral surface of the soft palate then 2 ml of (lidocaine HCL 2% and epinephrine 1:100000) was injected in the center of the soft palate and in 2 points paramedian to the uvula, then the patient was worn a protective laser eye glasses as well as the medical staff provided with proper laser safety measures. The diode laser (commercial trade market Diomed 15) which is Gallium, aluminum, arsenide (Ga AlAs) emits a wavelength of 810nm +20nm was delivered into six points on the oral surface of soft palate was identified for channeling (two in center of the soft palate near the uvula base and two paramedian near the junction of the soft and hard palate on each side. The laser fiber was 400 micron coupled to a surgical hand-piece with a curved tip was inserted interstitially into oral layer of the soft palate. The diode laser device was fixed on constant power at 6 watt with variable time between 7- 9 second. 

The total energy that given to whole of the soft palate should not exceeded the 1000 joule to generate temperature approximately 60-90 c enough for inducing localized thermo coagulation to produce scarring of the soft palate that will lead to fibrosis and increase the stiffness of the soft palate and decrease it's fluttering. Then the patient was able to be discharged after 1 hour on oral antibiotic as Amoxillin-Clavinic acid tablet 625 mg three times daily and oral analgesia as acetaminophen tablet 500mg three times daily, and mouth gargle three times daily for 7 days. Then follow up was arranged at 1 week, 1 month, 3 months and 6 months post-operative. The assessment of snoring intensity by VAS, frequency and duration by snoring severity scale was done. The same schedule of follow up was applied of Muller grading by flexible nasopharyngoscopy and the final assessment of both was at evaluated at six months post-operative.

*Statistical analysis*

Data analyzed using STATA software version 14. Continuous data represented by mean, standard deviations, Minimum and maximum. Categorical data represented by frequencies and percentages. Repeated analysis of variances (ANOVA) used to assess effect of diode laser on scoring snoring. P<0.05 was considered significant.

## Results

There were 34 (73.91%) males, and 12 (26.08%) females, with mean age of 45.31 (± 11.746) years. The average value of the Epworth sleepiness scale was 5.4±1.7SD for both gender; 5.1 ± 1.8 SD in males, and in 6.0 ±1.1 SD females, with P value of 0.182, while the mean value of body mass was 27.3±1.6 SD in males, and 27.7±0.9 SD in females with P value of 0.435, and for the neck circumference was 41.6±1.5 SD in males, and 37.0±1.5 SD in females, with P value <0. 001. 

**Fig 1:**
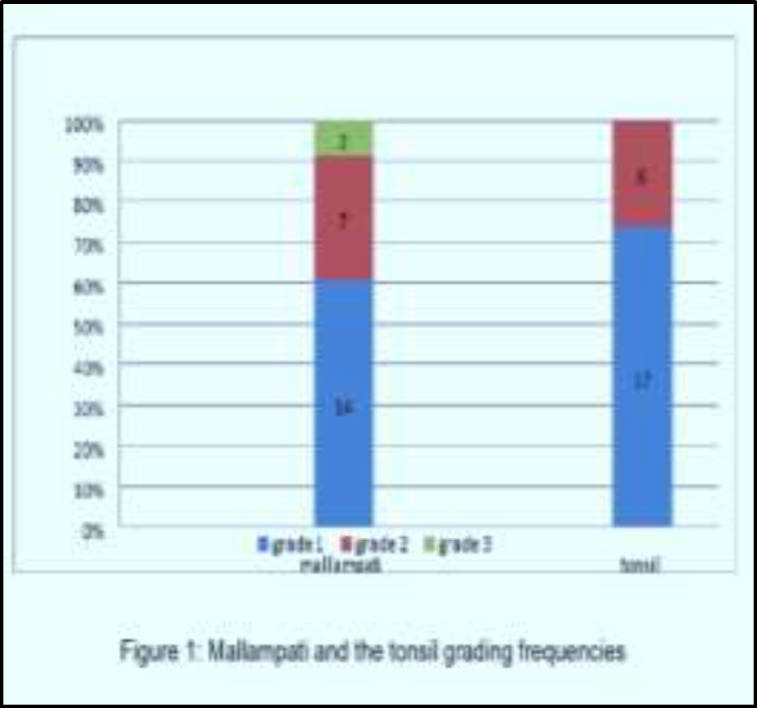
Mallampati and the tonsil grading frequencies

The frequency of the mallampati and the tonsil grading revealed, that (60.86 %) had mallampati grade 1, and about (73.91%) had tonsil grade 1, and these observations were highlighted in figure 1.The mean snoring intensity and the Muller maneuver scores across time of follow up for patients treated by Diode laser palatoplasty were revealed a significant improvement as the p value were < 0.05, table 2 showed these findings in detail.

**Table 2:** Mean score of snoring intensity and the Muller maneuver

**Parameters**	**Average scores**						**Effect**
	**Pre-op**	**1 week** **post op**	**1 month** **post op**	**3 months** **post op**	**6 months** **post op**		
Snoring intensity	6.6 (P =0.89)	6.1 (P = 0.045)	3.3 (P= 0.035)	2.5 (P = 0.018)	2.4 ( P=0.001)		92.1%
Muller maneuver	2.7 (P = 0.76)	2.6 (P = 0.048)	2.4 (P = 0.026)	2.0 (P = 0.013)	1.9 (P =0.001)		81.1%

In current study we found that 9 patients were complained from loud snoring VAS (4-6) at a baseline, eight of them were improved to mild snoring (1-3), and one patient get totally improved (no snoring), while 14 patients were complained from very loud snoring (7-9) at the base time, five of them were had some improvement as their snoring score grade was declined to loud grade (4-6), seven of them whom snoring scoring grade were decline to mild grade (1-3), however only one patient get no benefit from the surgery. 

So the overall frequencies of improvement of snoring loudness results had revealed a total improvement (no snoring) were detected in 4 patients, and moderate improvement had been found in 30 patients, while mild improvement of snoring had been seen in 10 patients, however about 2 patients got no benefit from the surgery.

**Fig 2:**
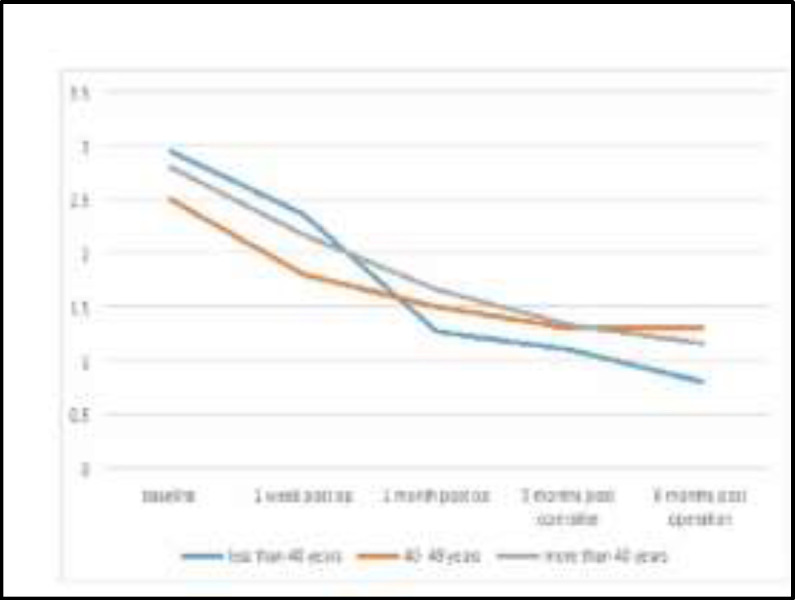
Age groups and snoring loudness

The correlation of the snoring loudness with some clinical parameters such as the age, gender, the mallampati grading, as well as the relation with its frequency and duration with were studied and revealed the following results; 

 In regard to the patients age groups; it showed the age group below 40 years had best clinical response to surgery as the score declined from 2.95 preoperatively to 0. 8 postoperatively, as it shown figure 2.

**Fig 3:**
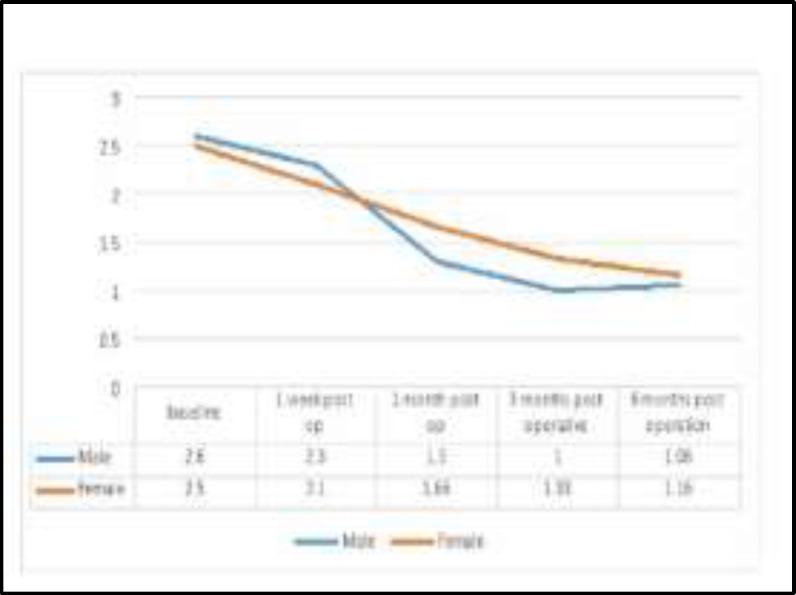
Gender and loudness

Regarding the correlation between the snoring loudness with the gender revealed that it in males the score was improved from 2.6 preoperatively to 1.06 postoperatively and in females from 2.5 to 1.16 postoperatively, as it shown in figure 3.

**Fig4:**
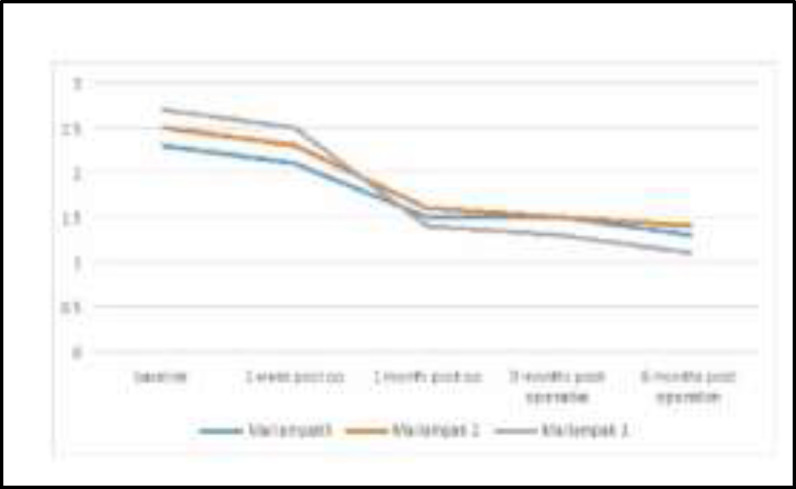
Mallampati grading and loudness of snoring

The correlation between the mallampati grading with snoring loudness were shown an obvious improvement various gradings, however, mallampati grade 1 showed to be had the best clinical response to surgery as its score was declined from 2.7 to 1.1, as it shown in figure 4.

**Fig 5:**
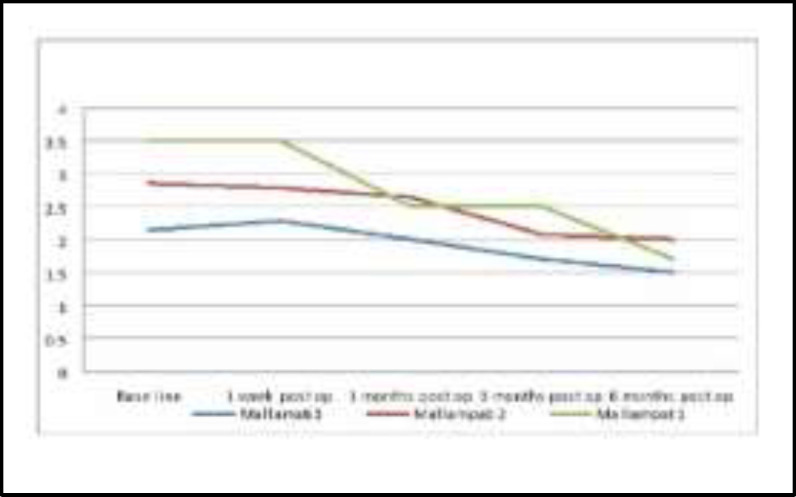
Mallampati and Muller Maneuver

Also the correlation between various mallampati grading and the Muller maneuver had shown a significant improvement (p value= 0.001), particularly grade 1 from score of 3.5 preoperatively to score 1.7 postoperatively, as it shown in figure 5.

**Fig 6:**
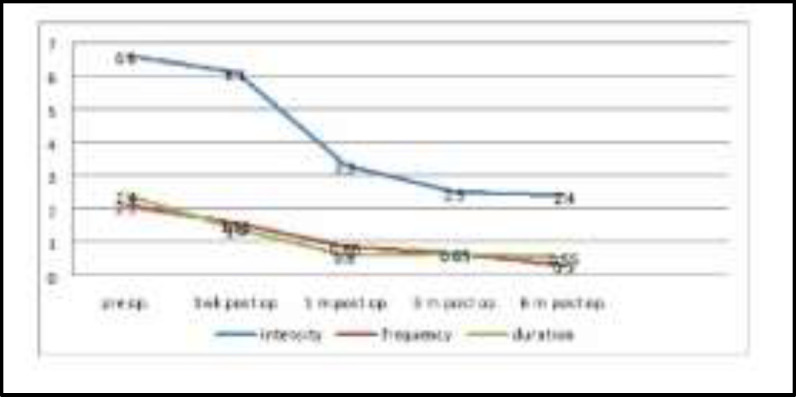
Snoring loudness and the frequency and duration

The snoring loudness showed a significant improvement as its score had been declined from 6.6 to 2.4, and for frequency (2.1 Hz to 0.3 Hz), while for duration (2.4 to 0.55), as the p values were from .075 to 0.023 respectively, as shown in figure 6.

## Discussion

Snoring may impact breathing during sleep and may lead to many socially troublesome problems, as well as it serves as a hazard factor for development of sleep apnea and its cardiopulmonary consequences, therefore several surgical options for snoring have innovated in the past years especially with the advent of minimally invasive office procedures such as laser palatoplasty which can be performed under local anesthesia in the office with high degree of patient expectations in relieving of snoring specially without the presence of OSA and with minimum post-surgical morbidities (9). In current study the mean patients age was 45.31 ± 11.746 year and, with males predominance, and the BMI of both gender was 27.4± 1.5, and these results were almost comparable with a study done by Hukins CA et al (10), as they showed the average age of their patients was 43.2 ± 11.1 year. These finding were depend upon patient life style and their tissue characteristics.

Selection of patient has been extensively investigated regarding to this sort of palatal surgery as they should meet precise and highly restricted inclusion criteria.

 Regarding the mean NC of both genders was 40.4 ± 2.5, while in Kim SE et al (11) study revealed the mean NC of both gender was 37.9 ± 3.4 cm, and also the Epworth sleepiness score was assessed and it showed that in the current study the average of Epworth sleepiness score of at the baseline of both gender was 5.4 ± 1.7, also in study done by Hukins CA et al (10), showed the average of Epworth sleepiness score at the baseline was 8.1±4 these based upon eligible patient body character to avoid any confounding factors that might interfere with the strict indication to surgery.

A population based cohort studies showed that age, male gender, obesity (BMI), neck circumference, were considered as independent risk factors (12,13).

Snoring intensity evaluated and revealed the average of snoring intensity assessed by VAS reduced was 6.6 at the baseline to 2.4 at 6 months after operation, (p-value = 0.001) .This was in agreement with Main C et al (14), they found that the average of snoring intensity VAS assessment was reduced from 6.5 – 8.4 to 2.75 – 5.2. The improvement of snoring loudness result in current study was revealed that (47.82%) had a significant improvement, mild improvement in (10.86%), and moderate improvement in (36.95%), while no improvement in (4.34%), these results were almost comparable with Krespi YP and Kizhner V study, whom they found that (47%) had a significant improvement (15), some improvement in (31%), and then (15%) had mild improvement, while in about (7%) had no change. In current study we found that the average of Muller maneuver grading reduced significantly from 2.7 at the baseline to 1.9 (p value = 0.001), in comparison with a study done by Utley DS, Shin EJ, et al (16) show Muller maneuver grading significantly decreased from 2.5 ± 1.3 to 0.9 ± 1.3 (P<0.001). While in Marchese D research (17), they studied the anterior palatoplasty effectiveness and simple snoring found that Muller maneuver grading was reduced from 2.6 to 1.5. 

Although, a wake nasaopharyngoscopy with Muller maneuver is the assessment is the currently applied method applied, yet its beneficial in planning for surgery with the aim of correlating its results with surgical outcome the success expectations rate is still a matter of debate, as claimed that the upper airways obstruction assessment during the daytime is different from those that occur at night during sleep, also it do not quantify the negative pressure created by the patient, so in order to minimize these biases, a sleep induction videonasolaryngoscopy have been recommended instead, however, it is may be a difficult procedure as well as not always be convenient for generalized screening of patients being referred to palatal surgery (18).

This could explain the variability in the clinical response to this treatment modality, so patients with snoring due to non-palatal origin would not be expected to get benefit with palatal stiffening technique (19). Therefore a precise patient's selection with snoring originated solely by palatal flutter will take in consideration, as they will attend the best surgical outcome.

The frequency of mallampati grading was 60.86 % of grade 1, the tonsil grade 1 was 73.91%, also mallampati grade 1 was better clinical response to surgery in regard to loudness, and a study done by Rodrigues MM et al (20), they concluded that high mallampati score is an important isolated risk factor for the worsening of the apnea, also Liistro et al (21) study showed that a high mallampati score (grade 3 or 4) is associated risk factor for OSA. A Cahali BM et al (22), in their study concluded that, there is a good correlation between adult snorers and tonsil grading and objective tonsil volume. 

So several studies demonstrated that a high relationship between obstructive sleep breath disorder (snoring) and some anatomical variation of upper airway such as; high grade mallampati level, tonsil size, and the tongue base size (23).

Friedman M, et al (24) study, they grading the oral cavity and oropharynx into 4 stages as the following; stage 1; Tonsil size (1 to 4), stage 2; a modification of the mallampati classification (1 to 4), and stage 3; (>or<BMI of 40 kg/m2), and stage 4; a major craniofacial abnormalities, they concluded that, in stage I the success (80.6%,) in stage II (37.9%), while in stage III (8.1%). In "spite of they used (UPPP) rather laser palatoplasty" as a surgical technique for relieving of snoring".

The clinical parameters in relation to the snoring loudness (intensity) with other data were assessed and showed the following observation; age group <40 years had better clinical response to surgery even it was statistically non-significant (p=0.2) and this may reflect the general concept stated that "the tissue response to surgical intervention or trauma in younger age group is better than in elderly" (25). 

A study conducted by Larrosa F, et al (26) they found that there are non-significant differences in the surgical outcome in relation to various age groups.The average of both snoring duration and its frequency were assessed by snoring sleepiness scale were showed a significantly declination in frequency score from 2.1 to 0.3 for the and from 2.4 to 0.55 for the duration (P<0.001), a study performed by Littner M, et al revealed that snoring frequency and severity were both reduced by laser assisted uvulopalatoplasty (27).This study was conducted a prospective surgical technique for primary snoring without OSA that addresses palatal snoring through interstitial palatal laser stiffening, with minimum postoperative morbidities such as wound contamination, post-operative throat discomfort with associated dysphagia and dry throat, as the laser beam achieves an exact energy deliverance with an easy hand-piece design, optimizing this will assist those selected patients in relieving from the problem that may have impact on their quality of life.

However there were several limitations in this study such as; the unavailability of polysomnography, sleep video-nasoendoscopy, and acoustic analysis for snoring.

## Conclusion

Stiffening of the soft palate by diode laser had shown to be significantly beneficial in relieving the simple snoring intensity as it had a positive impact on its subjective loudness, frequency and duration. 
